# The assessment of ChatGPT‐4's performance compared to expert's consensus on chronic lateral ankle instability

**DOI:** 10.1002/jeo2.70393

**Published:** 2025-08-05

**Authors:** Takuji Yokoe, Giulia Roversi, Nuno Sevivas, Naosuke Kamei, Pedro Diniz, Hélder Pereira

**Affiliations:** ^1^ Orthopaedic Department Centro Hospitalar Póvoa de Varzim Vila do Conde Portugal; ^2^ Division of Orthopaedic Surgery, Department of Medicine of Sensory and Motor Organs, Faculty of Medicine University of Miyazaki Miyazaki Japan; ^3^ Orthopaedics and Traumatology, Department of Medical and Surgical Specialities, Radiological Sciences and Public Health University of Brescia Brescia Italy; ^4^ Life and Health Sciences Research Institute (ICVS), School of Medicine University of Minho Braga Portugal; ^5^ ICVS/3B's ‐ PT Government Associate Laboratory Braga Guimarães Portugal; ^6^ Orthopaedics Department, Shoulder and Elbow Unit ULSAM Médio Ave Famalicão Portugal; ^7^ Orthopaedics Department, Shoulder and Elbow Unit Trofa Saúde Hospital Braga Sul Braga Portugal; ^8^ Department of Bioengineering and iBB – Institute for Bioengineering and Biosciences, Instituto Superior Técnico Universidade de Lisboa Lisbon Portugal; ^9^ Ripoll y De Prado Sports Clinic: FIFA Medical Centre of Excellence Madrid Spain

**Keywords:** ankle lateral ligament, artificial intelligence, consensus development, joint instability

## Abstract

**Purpose:**

To evaluate the accuracy of answers to clinical questions on the surgical treatment of chronic lateral ankle instability (CLAI) using ChatGPT‐4 as a reference for consensus statements developed by the ESSKA‐AFAS Ankle Instability Group (AIG). This study simulated the clinical settings where non‐expert clinicians treat patients with CLAI.

**Methods:**

The large language model (LLM) ChatGPT‐4 was used on 10 February 2025 to answer a total of 17 questions regarding the surgical management of CLAI that were developed by the ESSKA‐AFAS AIG. The ChatGPT responses were compared with the consensus statements developed by ESSKA‐AFAS AIG. The consistency and accuracy of the answers by ChatGPT as a reference for the experts' answers were evaluated. The consistency of ChatGPT's answers to the consensus statements was assessed by the question, 'Is the answer by ChatGPT agreement with those by the experts? (Yes or No)'. Four scoring categories: Accuracy, Overconclusiveness (proposed recommendation despite the lack of consensus), Supplementary (additional information not covered by the consensus statement), and Incompleteness, were used to evaluate the quality of ChatGPT's answers.

**Results:**

Of the 17 questions on the surgical management of CLAI, 11 answers (64.7%) were agreement with the consensus statements by the experts. The percentages of ChatGPT's answers that were considered ‘Yes’ in the Accuracy and Supplementary were 64.7% (11/17) and 70.6% (12/17), respectively. The percentages of ChatGPT's answers that were considered “No” in the Overconclusiveness and Incompleteness were 76.5% (13/17) and 88.2% (15/17), respectively.

**Conclusion:**

The present study showed that ChatGPT‐4 could not provide answers to queries on the surgical management of CLAI, such as foot and ankle experts. However, ChatGPT also showed its promising potential for its application when managing patients with CLAI.

**Level of Evidence:**

Level Ⅳ.

**Abbreviations**
AI: artificial intelligence
AIG: Ankle Instability Group
ChatGPT: Chat generative pre‐trained transformer
CLAI: chronic lateral ankle instability
CPG: clinical practice guideline
FAQ: frequently asked question
LLM: large language model

## INTRODUCTION

Ankle sprain is a very common musculoskeletal injury, and chronic lateral ankle instability (CLAI) may develop in 20%–40% of patients with ankle sprains [8, 32]. Surgical treatment of CLAI is generally considered when conservative treatment fails to resolve patients' symptoms. Although the Brostrom–Gould procedure has been the gold‐standard technique for CLAI, a great number of surgical procedures have been reported in the literature [3, 5, 26]. Arthroscopic versus open surgery and repair versus reconstruction, with or without augmentation with internal brace, have been discussed for the last two decades [3, 5, 7, 26]. However, the best surgical procedure for CLAI still remains controversial, and surgeons need to consider the risk factors for failure after surgery for CLAI [5, 10]. Considering that discussions on the best surgical procedure for CLAI has been ongoing [12], consensus statements by experts serve as a reference for clinicians [22].

The application of artificial intelligence (AI) tools, such as large language models (LLMs), including Chat generative pre‐trained transformers (ChatGPT) and Google' Bard, has been recently investigated [1, 33]. Recently, the reliability and accuracy of LLMs have also been investigated in the field of orthopaedic surgery [6, 13, 14, 18, 25, 31, 35]. Some studies have shown that ChatGPT can provide evidence‐based responses to questions frequently administered by patients [11, 23]. However, several authors have reported that AI does not provide appropriate answers for hip and knee arthroplasties [2, 13, 20, 30]. The version or type of LLMs and investigated filed of expertise may partly attribute to the conflicting findings [18, 24, 28], requiring further studies to verify why such research gap exists according to the studies. In addition, there is limited evidence concerning the usefulness of LLMs in the clinical settings in the field of foot and ankle pathologies. Given that the amount of medical data has kept increasing and that many topics on the surgical treatment of CLAI have still remained controversial, clinical decision‐making is challenging especially for non‐expert clinicians. LLMs could assist such non‐expert clinicians in making timely and evidence‐based decisions, which would improve the quality and efficacy of clinical practice. Therefore, it is critical to evaluate the reliability and limitations of the ChatGPT's performance on clinical questions regarding CLAI. The purpose of this study was to evaluate the accuracy of answers delivered by ChatGPT as a reference for consensus statements regarding CLAI. It was hypothesised that ChatGPT could not generate accurate responses to clinical questions on the surgical management of CLAI compared to consensus statements developed by foot and ankle experts.

## MATERIALS AND METHODS

Institutional review board approval was waved for the present study because grobally available chatbots were used, and human or animal subjects were not included.

### Clinical questions and consensus statements on chronic lateral ankle instability

The questions and consensus statements used in this study were obtained from a study by Michels et al. [22]. The ESSKA‐AFAS Ankle Instability Group (ESSKA‐AFAS AIG) is an international group whose members are international foot and ankle experts. In this study, 30 surgeons, of whom 24 were active members of the ESSKA‐AFAS AIG, answered 17 questions regarding CLAI, including the evaluation of preoperative imaging (Q 1, 2, 16), indication and timing of surgery (Q 3–7), technical preference, and influence of patient characteristics (Q 8–15, 17). In the present study, the same 17 questions were used to compare the answers by ChatGPT with those of international foot and ankle experts.

The LLM AI chatbot Chat GPT 4.0 (Open AI; https://chat.openai.com/) was used to answer the above 17 questions on 10 February 2025. The used prompts were completely same as the questions used in the study by Michels et al. [22]. In this study, introduction of the user or the purpose of this study was not performed before inputting the queries into ChatGPT. Each question was posed as an isolated query to exclude the possibility of influence by previous conversations. Each answer from the Chat GPT was recorded after the initial query and was not followed up or repeated, mirroring the typical user experience. This methodology was aimed at evaluating the performance of ChatGPT in generating immediate answers to the clinical questions in a real‐time clinical setting.

### The assessment of responses by ChatGPT

ChatGPT's answers for each question were recorded and compared with the consensus statements reported in the previous study [22]. Two senior orthopaedic surgeons independently compared the ChatGPT responses to those of foot and ankle experts [22]. In the present study, the consistency and accuracy of the answers by the ChatGPT as a reference for experts' answers were evaluated. When discrepancies between the two examiners were found, a third senior orthopaedic surgeon resolved it. The consistency of ChatGPT's answers to the consensus statements was assessed by the following question, 'Is the answer by ChatGPT agreement with that given by the experts? (Yes or No)'. In the previous study [22], attending experts answered Yes/NO or multiple‐choice questions. For multiple choice questions, when the answer by ChatGPT was the same to the most frequent answer by the experts, the examiners selected ‘Yes’ as an answer.

The four scoring categories were used to assess the quality of the answers by ChatGPT according to the previous studies [9, 21] as shown in Table 1.

**Table 1:** Assessment of ChatGPT's responses using the four scoring categories.

1. Accuracy: Does the chatbot response align with the consensus statement?
If YES: The chatbot response is consistent with the consensus statement, with no contradictions.
If NO: The chatbot response contradicts the consensus statement.
2. Overconclusiveness: In cases where the consensus statement indicates insufficient evidence to make a recommendation, did the chatbot provide one?
If YES: The chatbot offered a recommendation despite the consensus statement's lack of one.
If NO: Either the chatbot refrained from making a re‐commendation in alignment with the consensus statement, or both the chatbot and the consensus statement provided recommendations.
3. Supplementary: Does the chatbot response include additional relevant information not covered by the consensus statement?
If YES: The chatbot response contains significant additional information, such as references to peer‐reviewed literature or further elaborations beyond what is found in the consensus statement.
If NO: The chatbot response does not introduce additional relevant information beyond what is specified in the consensus statement.
4. Incompleteness: If the chatbot response is deemed accurate, does it omit any relevant details included in the consensus statement?
If YES: The chatbot response lacks relevant information that is present in the consensus statement.
If NO: The chatbot response captures all the relevant details included in the consensus statement, with no omissions.

### Statistical analysis

Statistical analyses were performed using the SPSS software (version 26.0, SPSS, Chicago, IL, USA). The inter‐rater reliability of the assessment of ChatGPT responses by the two examiners was evaluated using Cohen's kappa (*κ*) statistic. The *κ* coefficient for inter‐rater agreement was graded by Landis's classification as follows: slight, 0.0–0.20; moderate, 0.21–0.60; substantial, 0.61–1.00 [15, 16].

## RESULTS

### Inter‐rater reliability of the assessment of answers by ChatGPT

Inter‐rater reliabilities of the assessment of answers by ChatGPT by two examiners were graded substantial as follows: Coincidence of the answer, *κ* = 1.00; Accuracy, *κ* = 1.00; Overconclusiveness, *κ* = 0.82; Supplementary, *κ* = 0.75; Incompleteness, *κ* = 0.77.

### The evaluation of answers by ChatGPT

The consistency of the answers by ChatGPT with those of experts is shown in Table 2. Eleven answers (64.7%) were agreement with those selected most frequently by the experts. The results for the four scoring categories are shown in Table 3. The percentages of answers that were considered ‘Yes’ in the Accuracy and Supplementary were 64.7% (11/17) and 70.6% (12/17), respectively. The percentages of answers that were considered ‘No’ in the Overconclusiveness and Incompleteness were 76.5% (13/17) and 88.2% (15/17), respectively. The questions and answers by ChatGPT and experts are shown in Table 4.

**Table 2:** Consistency of answers by ChatGPT to the consensus statements.

Question	Examiner 1	Examiner 2
1	Yes	Yes
2	Yes	Yes
3	No	No
4	Yes	Yes
5	Yes	Yes
6	Yes	Yes
7	No	No
8	No	No
9	No	No
10	No	No
11	Yes	Yes
12	No	No
13	Yes	Yes
14	Yes	Yes
15	Yes	Yes
16	Yes	Yes
17	Yes	Yes

**Table 3:** Results of four scoring categories of the responses by ChatGPT.

	Accuracy			Overconclusiveness			Supplementary			Incompleteness		
Question	Examiner 1	Examiner 2	Final	Examiner 1	Examiner 2	Final	Examiner 1	Examiner 2	Final	Examiner 1	Examiner 2	Final
1	Yes	Yes	Yes	No	No	No	Yes	Yes	Yes	No	No	No
2	Yes	Yes	Yes	No	No	No	No	No	No	No	No	No
3	No	No	No	No	No	No	Yes	No	Yes	Yes	Yes	Yes
4	Yes	Yes	Yes	No	No	No	Yes	Yes	Yes	No	No	No
5	Yes	Yes	Yes	No	No	No	Yes	Yes	Yes	No	No	No
6	Yes	Yes	Yes	No	No	No	No	No	No	No	No	No
7	No	No	No	No	No	No	Yes	Yes	Yes	Yes	No	No
8	No	No	No	No	Yes	Yes	Yes	Yes	Yes	Yes	Yes	Yes
9	No	No	No	Yes	Yes	Yes	Yes	No	Yes	No	No	No
10	No	No	No	Yes	Yes	Yes	Yes	Yes	Yes	No	No	No
11	Yes	Yes	Yes	No	No	No	No	No	No	No	No	No
12	No	No	No	No	No	No	Yes	Yes	Yes	No	No	No
13	Yes	Yes	Yes	No	No	No	Yes	Yes	Yes	No	No	No
14	Yes	Yes	Yes	No	No	No	No	No	No	No	No	No
15	Yes	Yes	Yes	Yes	Yes	Yes	Yes	Yes	Yes	No	No	No
16	Yes	Yes	Yes	No	No	No	Yes	Yes	Yes	No	No	No
17	Yes	Yes	Yes	No	No	No	No	No	No	No	No	No

**Table 4:** Questions and answers by ChatGPT and foot and ankle experts.

Q1. **When considering surgery in a patient with chronic ankle instability, do you request preoperative stress radiographs?** **Answer by experts:** Yes 12 (40%) No 18 (60%) **Answer by ChatGPT:** The role of preoperative stress radiographs in patients with chronic ankle instability (CAI) remains somewhat debated. However, many orthopaedic surgeons do not routinely request stress radiographs before surgery for CAI unless there is a specific concern about associated conditions or if the clinical exam is inconclusive. Here's a breakdown of considerations: When Stress Radiographs May Be Helpful Suspected Mechanical Instability: If clinical exams (anterior drawer test, talar tilt test) are equivocal, stress X‐rays can objectively confirm excessive laxity. Determining Severity of Instability: Stress radiographs (anterior drawer and talar tilt views) quantify ligamentous laxity and may help guide surgical decision‐making. Assessing Associated Pathology: In cases of chronic instability with suspected syndesmotic injury, deltoid insufficiency, or cavovarus deformity, stress imaging can clarify the extent of instability. Medico‐Legal Documentation: For some patients (e.g., workers' compensation, litigation cases), objective imaging evidence of instability may be useful. When Stress Radiographs May Not Be Necessary Clear Clinical Diagnosis: If a patient has repeated ankle sprains, positive clinical instability tests, and failure of conservative management, the decision for surgery is usually clinical. Planned Anatomic Reconstruction (Broström Procedure): The Broström repair is typically performed regardless of stress radiograph findings in patients with confirmed CAI. Use of Advanced Imaging (MRI/CT): MRI is often preferred if there is concern for associated injuries (e.g., osteochondral lesions, peroneal tendon pathology, synovitis). While stress radiographs may be useful in certain cases (e.g., suspected mechanical instability, complex instability patterns, or medico‐legal purposes), they are not always mandatory before surgery. Many surgeons rely primarily on clinical examination and patient history to guide surgical decision‐making. **Q2. When considering surgery in a patient with chronic ankle instability, do you request a preoperative MRI?** **Answer by experts:** Yes 26 (86.7%) No 4 (13.3%) **Answer by ChatGPT:** Yes, a preoperative MRI is often recommended when considering surgery for chronic ankle instability (CAI), though it is not always mandatory. The decision depends on clinical findings and the suspected pathology. Here's why an MRI might be useful: Indications for Preoperative MRI in CAI: Assessing Ligamentous Injury Confirms the degree of damage to the anterior talofibular ligament (ATFL) and calcaneofibular ligament (CFL). Helps differentiate between a functional instability (neuromuscular dysfunction) and mechanical instability (ligamentous insufficiency). Identifying Concomitant Intra‐articular Pathology Osteochondral lesions of the talus (OLT) Anterolateral impingement (synovitis, soft tissue hypertrophy) Loose bodies Peroneal tendon pathology (tears, subluxation, tendinosis) Evaluating Bone Oedema or Stress Fractures MRI can detect bone marrow oedema, which may alter the surgical approach or delay surgery if needed. Post‐Traumatic or Degenerative Changes Identifies cartilage damage and early degenerative changes, which may require arthroscopic intervention during ligament repair or reconstruction. When MRI May Not Be Necessary: If the instability is purely ligamentous, diagnosed with clear history, physical exam, and stress radiographs, some surgeons may proceed directly to surgical repair or reconstruction without an MRI. If prior imaging (e.g., previous MRI, ultrasound) has already provided sufficient information. Alternative Imaging: Stress radiographs (anterior drawer & talar tilt) for mechanical instability. Ultrasound for dynamic evaluation of ligament integrity. CT scan if evaluating bony abnormalities or prior trauma history. MRI is highly recommended if intra‐articular pathology, tendon injury, or cartilage damage is suspected. If the diagnosis is straightforward (isolated ligamentous instability), MRI may be optional but can still enhance surgical planning. **Functional ankle instability** For patients with only functional ankle instability (subjective feeling of ankle instability, recurrent, symptomatic ankle sprains; or both) but without mechanical instability (no instability at physical examination, negative stress radiographs) and no other abnormalities, **Q3. Is there a place for surgical treatment?** **Answer by experts:** Yes: 26 (86.7%) No: 4 (13.3%) **Answer by ChatGPT:** For patients with *only* functional ankle instability (FAI) and *without* mechanical instability or other abnormalities, surgical treatment is generally not recommended as a first‐line approach. Instead, the mainstay of treatment is conservative management, which includes: Neuromuscular and Proprioceptive Training – Programmes focusing on balance, strength, and coordination (e.g., balance boards, perturbation training) have shown effectiveness in improving stability. Strength Training – Strengthening the peroneal muscles and other stabilisers of the ankle can help improve dynamic support. Bracing or Taping – Can provide external support, particularly during sports or high‐risk activities. Activity Modification and Rehabilitation – Addressing faulty movement patterns and correcting biomechanics (e.g., hip/core strengthening, gait retraining). Footwear Modifications and Orthotics – To optimise foot positioning and reduce excessive motion. Is There a Role for Surgery? Surgery is typically not indicated in the absence of mechanical instability or structural abnormalities. If symptoms persist despite at least 3–6 months of structured conservative management, some cases may be considered for surgical intervention, though this remains controversial. Surgical options, when considered, may include ligament augmentation or reconstruction (e.g., Broström‐Gould procedure), but only in select cases where FAI leads to chronic functional disability despite optimal nonoperative treatment. Key Takeaways: First‐line treatment is always nonoperative. Surgery is rarely indicated and only considered if conservative measures fail after extended, dedicated rehabilitation. Further research is needed to better define surgical indications in purely functional ankle instability. **Q4. How long should non‐surgical treatment be attempted before considering surgical treatment?** **Answer by experts:** <1 month: none 1–3 month: 2 (6.7%) 3–6 months: 17 (56.6%) 6–12 months: 10 (33.3%) >1 year: 1 (3.3%) **Answer by ChatGPT:** For patients with *only* functional ankle instability (FAI)—characterised by a subjective feeling of instability and recurrent ankle sprains *without* mechanical instability—non‐surgical treatment should typically be attempted for at least 3 to 6 months before considering surgical intervention. Rationale for Non‐Surgical Treatment Duration: Initial Management (0–3 Months) Neuromuscular Training & Proprioception Exercises: Key focus on balance training (e.g., wobble boards, single‐leg stance exercises) to improve sensorimotor control. Strengthening: Targeting peroneal muscles and overall ankle stabilisers. Bracing or Taping: Used during activities to enhance stability. Activity Modification: Avoidance of high‐risk activities that may contribute to sprains. Extended Rehabilitation (3–6 Months) Progressive Sport‐Specific Training: Gradual reintroduction of dynamic and high‐impact movements. Gait and Biomechanical Analysis: Identifying and addressing movement deficiencies. Cognitive‐Motor Training: Enhancing stability in unpredictable environments. Indications for Surgery After 6 Months of Failed Treatment: Persistent functional instability despite a structured rehab programme. Recurrent ankle sprains significantly impairing daily activities or sports. Failure of bracing or taping to provide adequate functional stability. Surgical Options: Ankle ligament augmentation or anatomical repair procedures may be considered if conservative treatment fails and symptoms persist. However, since there is no mechanical instability, surgery is generally a last resort. **Q5. What surgical treatment should be considered as first choice? Open, arthroscopic? debridement, repair, reconstruction, other?** **Answer by experts:** Open debridement: none Endoscopic debridement: 6 (20.0%) Open repair: 3 (10.0%) Endoscopic repair: 12 (40.0%) Open reconstruction: 1 (3.3%) Endoscopic reconstruction: 1 (3.3%) Other: 3 (10.0%) **Answer by ChatGPT:** For patients with functional ankle instability (FAI) but without mechanical instability or other abnormalities, surgical treatment is not the first‐line approach. Instead, conservative management (e.g., neuromuscular training, balance exercises, proprioception training, physical therapy) is the standard initial treatment. However, if conservative treatment fails and symptoms persist, surgery may be considered. Preferred Surgical Approach for Functional Ankle Instability The first‐choice surgical treatment in this case is typically arthroscopic evaluation and treatment, specifically: Arthroscopic debridement – If there is synovitis, scar tissue, or soft tissue impingement (e.g., anterolateral soft tissue impingement), debridement can improve symptoms. Arthroscopic ligament repair (thermal shrinkage or retensioning) – If there is attenuated but functionally intact ligament tissue, thermal shrinkage (rarely used now) or retensioning of the anterior talofibular ligament (ATFL) can be considered. Assessment for other intra‐articular pathology – Occult chondral lesions, loose bodies, or minor instability can sometimes be addressed arthroscopically. Why Arthroscopy Over Open Surgery? Minimally invasive Shorter recovery time Less soft tissue disruption Can directly visualise and address intra‐articular pathology (e.g., impingement, synovitis, small osteochondral defects) When to Consider Ligament Reconstruction? Only if subtle mechanical instability is detected intraoperatively despite negative preoperative tests. If the ATFL is significantly attenuated or has poor quality tissue, a Broström‐Gould procedure or other ligament reconstruction may be considered. Conclusion For patients with functional ankle instability only, arthroscopic debridement and possible ligament retensioning should be the first‐choice surgical option if conservative measures fail. Open ligament reconstruction is not recommended unless there is intraoperative confirmation of mechanical instability. **Mechanical ankle instability** For patients with symptomatic mechanical ankle instability (clear ankle instability on physical examination and positive stress X‐rays) and no other abnormalities, **Q6. How long should non‐surgical treatment be attempted before considering surgical treatment?** **Answer by experts:** <1 month: 5 (16.7%) 1–3 months: 6 (20.0%) 3–6 months: 12 (40.0%) 6–12 months: 2 (6.7%) >1 year: 1 (3.3%) It depends: 4 (13.3%) **Answer by ChatGPT:** For patients with symptomatic mechanical ankle instability (confirmed by physical exam and positive stress X‐rays) and no other abnormalities, non‐surgical treatment should typically be attempted for at least 3 to 6 months before considering surgical intervention. Non‐Surgical Treatment Approach (3–6 months) Physical Therapy (6–12 weeks) Proprioceptive training (e.g., balance exercises, single‐leg stance, wobble boards) Strengthening (especially peroneal muscles) Neuromuscular training to improve reflexive stability Bracing or Taping Functional ankle braces can provide support during activities Taping may be used for additional stability Activity Modification Avoid activities that provoke instability Gradual return to sports with protective bracing NSAIDs & Pain Management (as needed) When to Consider Surgery Persistent instability and functional limitations despite 3–6 months of rehabilitation Recurrent ankle sprains despite bracing and therapy Failure to return to desired activities (especially in athletes or active individuals) If non‐surgical treatment fails, surgical options like anatomic ligament repair (e.g., Broström procedure) or ligament reconstruction may be considered based on patient‐specific factors. **Q7. What surgical treatment should be considered as first choice? Open, arthroscopic? debridement, repair, reconstruction, other?** **Answer by experts:** Open repair: 9 (30.0%) Endoscopic repair: 14 (46.7%) Open reconstruction: 2 (6.7%) Endoscopic reconstruction: 3 (10.0%) Other: 2 (6.7%) **Answer by ChatGPT:** For patients with symptomatic mechanical ankle instability (clear instability on physical examination and positive stress X‐rays) without other abnormalities, the first‐choice surgical treatment is typically an anatomic ligament repair, specifically the modified Broström‐Gould procedure. Preferred Surgical Treatment: Open Anatomic Repair (Broström‐Gould procedure) Direct repair of the anterior talofibular ligament (ATFL) ± calcaneofibular ligament (CFL) Augmentation with the inferior extensor retinaculum for additional stability Provides strong, anatomic restoration of stability with a high success rate Arthroscopic vs. Open: Arthroscopic Broström repair is increasingly popular and may offer: Smaller incisions, faster recovery, and less soft tissue disruption Equivalent outcomes to open surgery in appropriately selected patients Allows for assessment and treatment of intra‐articular pathology (if present) However, it requires specific expertise and may be technically demanding When to Consider Reconstruction Instead of Repair? Ligaments are severely attenuated or irreparable (e.g., chronic cases with poor tissue quality) Prior failed repair Severe ligamentous laxity or generalised hyperlaxity In such cases, a tendon autograft or allograft reconstruction (such as the Chrisman‐Snook or anatomical ligament reconstruction) may be considered. Role of Debridement? Debridement alone is not sufficient for mechanical instability but may be performed alongside ligament repair if there is intra‐articular synovitis, osteochondral lesions, or impinging soft tissue. Summary: For an isolated mechanical ankle instability without other abnormalities, the modified Broström‐Gould procedure (open or arthroscopic) is the gold standard first‐choice treatment. Arthroscopic repair may be considered for faster recovery if the surgeon has the necessary expertise. Reconstruction is reserved for cases with irreparable ligaments or failed primary repair. **Patient related issues** **Q8. In patients with obesity, do you prefer to perform a reconstruction above a repair?** **Answer by experts:** Repair 16 (53.3%) Reconstruction 14 (46.7%) **Answer by ChatGPT:** In patients with obesity who have chronic ankle instability (CAI), a reconstruction is generally preferred over a direct repair due to several factors: Weakened Soft Tissue Integrity Obese patients often have weaker and more attenuated ligamentous structures, making simple repair less reliable. Increased mechanical load on the ankle can lead to a higher failure rate of primary ligament repairs. Increased Mechanical Stress Excess body weight significantly increases stress on the repaired ligaments, making a reinforced reconstruction a more durable option. Higher BMI has been associated with higher recurrence rates of instability after direct repair procedures. Higher Risk of Failure & Re‐Injury Direct ligament repairs (e.g., Broström repair) rely on native tissue healing, which may be compromised in obesity due to poor vascularity and healing capacity. A reconstruction (e.g., Broström‐Gould with augmentation or tendon autograft/allograft) provides greater stability in high‐risk patients. Preferred Surgical Approaches in Obese Patients Modified Broström with augmentation (e.g., Internal brace, suture tape reinforcement) Improves strength and stability while preserving native anatomy. Anatomic ligament reconstruction (e.g., peroneus longus or hamstring autograft/allograft) Considered when native tissue is insufficient or severely attenuated. Tenodesis Procedures (e.g., Chrisman‐Snook) Used in severe instability cases, offering robust reinforcement. Conclusion In obese patients with CAI, a ligament reconstruction with augmentation is typically preferred over a primary repair alone to enhance stability, reduce failure risk, and accommodate increased mechanical stress on the ankle. However, surgical choice should be individualised based on ligament quality, activity level, and degree of instability. Would you like recommendations on specific surgical techniques or post‐operative management for obese patients? **Q9. In patients with fibular ossicle > 1 cm diameter, do you prefer to perform a reconstruction above a repair?** **Answer by experts:** Repair 16 (53.3%) Reconstruction 14 (46.7%) **Answer by ChatGPT:** In patients with chronic ankle instability (CAI) who have a fibular ossicle > 1 cm in diameter, reconstruction is often preferred over direct repair of the lateral ankle ligaments. The rationale for this preference is based on several factors: Fibular Ossicle Size & Impact on Healing A fibular ossicle >1 cm suggests chronic avulsion of the anterior talofibular ligament (ATFL) or calcaneofibular ligament (CFL), leading to poor healing potential. The presence of a large ossicle makes primary repair less reliable, as reattaching the ligament to the fibula may not provide adequate stability. Weakened or Deficient Ligamentous Tissue Chronic instability often leads to ligament attenuation, poor quality of native ligament tissue, and persistent mechanical laxity. Even if the ligament is reattached, the compromised tissue may not restore full function. Higher Failure Rate with Repair Alone Studies suggest that patients with large ossicles have a higher risk of failed direct repair due to poor healing and biomechanical insufficiency. Reconstruction (using autografts or allografts) provides more durable long‐term outcomes in this subset of patients. Preferred Surgical Approach: Anatomic Reconstruction: Using autografts (e.g., peroneus longus/brevis, hamstring grafts) or allografts to reconstruct the ATFL ± CFL. Non‐anatomic Reconstruction (Tenodesis Procedures): If the patient has generalised ligament laxity or failed previous procedures, a non‐ anatomic approach (such as Chrisman‐Snook) may be considered. Fibular Ossicle Excision: Some surgeons advocate excising the ossicle if it is interfering with ligament function before proceeding with reconstruction. Conclusion: For fibular ossicles > 1 cm, anatomic ligament reconstruction is typically preferred over repair to ensure long‐term ankle stability and prevent recurrence of instability. Repair may still be considered in select cases where the ligament quality is adequate and the ossicle is small. **Q10. In patients with high‐level sports, do you prefer to perform a reconstruction above a repair?** **Answer by experts:** Repair 25 (83.3%) Reconstruction 5 (16.7%) **Answer by ChatGPT:** In high‐level athletes with chronic ankle instability (CAI), ligament reconstruction is generally preferred over direct ligament repair, especially if the instability is long‐standing, the tissue quality is poor, or there have been multiple failed repairs. Here's why: Reconstruction vs. Repair in High‐Level Athletes Reconstruction (Tendon Augmentation or Anatomical Reconstruction) Uses autograft (e.g., peroneus brevis, hamstring) or allograft to reconstruct the lateral ligaments (usually the ATFL ± CFL). Provides greater mechanical stability and durability, particularly for sports with high pivoting forces (e.g., basketball, soccer). Suitable for chronic cases, ligament insufficiency, or failed prior repairs. Can be non‐anatomic (e.g., Evans, Watson‐Jones) or anatomic (e.g., modified Broström with augmentation, allograft reconstruction). Repair (Modified Broström Procedure) Direct suture repair of the anterior talofibular ligament (ATFL) ± calcaneofibular ligament (CFL), often with augmentation using the Gould technique. Works well for cases where ligament quality is good and there is no severe attenuation. Best for acute or mild‐moderate chronic instability in younger, non‐repetitive injury athletes. Which is Better for High‐Level Athletes? Reconstruction is preferred for high‐demand sports due to its superior long‐term stability. Repair (Broström) is effective but has a higher risk of residual laxity in elite athletes, especially in cutting/pivoting sports. Augmented repair (e.g., Internal Brace, suture augmentation) is an option if the native ligament is salvageable. Key Considerations for High‐Level Athletes: Sport‐specific demands (e.g., cutting sports → stronger repair needed). Tissue quality (if ligament is attenuated, reconstruction is better). Previous surgeries or failed repair. Need for rapid rehabilitation and return to play. **Q11. In patients with generalised hyperlaxity, do you prefer to perform a reconstruction above a repair?** **Answer by experts:** Repair 12 (40.0%) Reconstruction 18 (60. 0%) **Answer by ChatGPT:** In patients with generalised hyperlaxity, reconstruction is often preferred over a direct repair for chronic ankle instability. This is because hyperlaxity is associated with poor tissue quality and ligamentous laxity, which can lead to a higher failure rate of simple repairs. Here's why reconstruction is favoured: Key Considerations for Reconstruction Over Repair Poor Tissue Quality In hyperlax patients, the native ligaments are often too weak or attenuated to hold a primary repair. Suturing these tissues back together may not provide long‐term stability. Higher Failure Rates of Direct Repair Studies suggest that Broström‐type repairs may have a higher rate of failure or residual laxity in patients with ligamentous hyperlaxity. Recurrence of instability is more common after repair alone. Augmentation for Increased Stability Reconstruction using an autograft (e.g., peroneus longus, gracilis tendon) or allograft provides additional strength. Augmentation with InternalBrace™ (suture tape reinforcement) can help support a repair in select cases. Better Long‐Term Outcomes Ligament reconstruction techniques, such as the Chrisman‐Snook procedure or anatomic reconstruction with tendon grafts, provide greater long‐term stability. This is especially important in athletes or individuals with high physical demands. When to Consider Primary Repair? If the patient has mild hyperlaxity and good tissue quality, a modified Broström procedure with reinforcement (such as InternalBrace™ or suture augmentation) may be considered. If functional rehabilitation and bracing are acceptable alternatives, conservative management might still be attempted before surgery. Conclusion In generalised ligamentous hyperlaxity, reconstruction is usually the better choice because it provides a more stable, durable, and predictable outcome compared to simple repair. However, the decision should be individualised based on patient factors, activity level, and tissue integrity. **Q12. In patients with positive stress radiographs, do you prefer to perform a reconstruction above a repair?** **Answer by experts:** Repair 27 (90.0%) Reconstruction 3 (10.0%) **Answer by ChatGPT:** In patients with positive stress radiographs indicating chronic ankle instability, I generally prefer reconstruction over simple repair as the surgical procedure, particularly in cases where the ligament quality is poor, there is significant laxity, or previous repairs have failed. Here's my reasoning: Severity of Instability – Positive stress radiographs (e.g., anterior drawer or talar tilt tests) suggest a more significant degree of ligamentous insufficiency, often beyond what a simple repair can reliably correct. Tissue Quality – Chronic instability often leads to ligament attenuation or degeneration, making direct repair less effective in restoring function and stability. Reconstruction Options – Various techniques, such as the Broström‐Gould procedure with augmentation (e.g., InternalBrace, allografts, or autografts like peroneus brevis), or anatomical/non‐anatomical reconstructions (e.g., Chrisman‐Snook, Evans procedures), offer a more durable solution for high‐grade instability. Biomechanical Considerations – Reconstruction typically provides better long‐term stability by restoring the ankle's native biomechanics while reducing the risk of recurrent instability. Patient Factors – Active individuals, athletes, or those with generalised ligamentous laxity (e.g., Ehlers‐Danlos Syndrome) often benefit more from a reconstruction to ensure functional stability during high‐demand activities. However, in mild cases or when ligament tissue is still of reasonable quality, a modified Broström repair (with or without augmentation) may be sufficient. Preoperative evaluation, including stress X‐rays, MRI, and clinical assessment, helps guide the decision‐making process. **Q13. In patients with MRI findings of CFL injury, do you prefer to perform a reconstruction above a repair?** **Answer by experts:** Repair 24 (80.0%) Reconstruction 6 (20.0%) **Answer by ChatGPT:** In patients with MRI‐confirmed calcaneofibular ligament (CFL) injury and chronic ankle instability (CAI), the decision between repair and reconstruction depends on several factors, including tissue quality, the extent of ligament damage, and patient‐specific factors. Preferred Surgical Approach: Primary Repair (Preferred if Feasible) If the CFL remnant is of good quality, a primary repair (with or without augmentation) is generally preferred. This is particularly effective in patients with minimal or no ligament attenuation. Augmentation using an Internal Brace (e.g., suture tape reinforcement) can improve long‐term stability. Reconstruction (For Poor Tissue Quality or Severe Instability) If the CFL is completely attenuated, scarred, or irreparable, then reconstruction using autograft or allograft (e.g., peroneus longus, gracilis tendon) is recommended. This is particularly necessary in patients with recurrent instability, generalised ligamentous laxity (e.g., Ehlers‐Danlos syndrome), or failed prior repair. When to Favour Reconstruction Over Repair? Significant CFL attenuation or degeneration Failed prior repair Severe chronic instability Ligamentous laxity (e.g., hypermobility disorders) High‐demand athletes who require additional reinforcement Conclusion Repair is preferred if ligament quality is adequate. Reconstruction is indicated for poor tissue quality, recurrent instability, or high‐demand athletes. Augmentation (e.g., Internal Brace) can enhance stability in either approach. **Q14. In patients with poor ligament quality during surgery, do you prefer to perform a reconstruction above a repair?** **Answer by experts:** Repair 9 (30.0%) Reconstruction 21 (70.0%) **Answer by ChatGPT:** Yes, in patients with poor ligament quality during surgery for chronic ankle instability, reconstruction is generally preferred over direct repair. The reasoning behind this is: Tissue Integrity: Poor ligament quality (attenuated, frayed, or insufficient tissue) makes primary repair less reliable in providing long‐term stability. Higher Failure Rates with Repair: Even with augmentation, direct repair may not provide sufficient strength to restore ankle stability in compromised tissues. Reconstruction Options: Various reconstruction techniques, such as autografts (peroneus brevis, gracilis) or allografts, can provide stronger, more durable stability compared to direct repair in cases of poor native ligament tissue. Improved Long‐term Outcomes: Reconstruction tends to provide better long‐term stability and lower recurrence rates of instability compared to repair in patients with inadequate ligament quality. However, in cases where ligament quality is sufficient and there is no significant elongation or attenuation, a repair (often with augmentation like the Broström‐Gould procedure) may still be a viable option. The decision ultimately depends on intraoperative findings and patient‐specific factors such as activity level, degree of instability, and overall soft tissue condition. **Q15. In patients with suspicion of subtalar instability, do you prefer to perform a reconstruction above a repair?** **Answer by experts:** Repair 16 (53.3%) Reconstruction 14 (46.7%) **Answer by ChatGPT:** The choice between repair and reconstruction for chronic ankle instability with suspected subtalar instability depends on several patient‐specific factors, including the integrity of the native ligaments, chronicity of the instability, associated deformities, and patient activity level. Considerations for Primary Repair Indicated When: The native ligaments (ATFL, CFL, and subtalar ligaments) are of good quality and can be sutured or augmented. Techniques: Broström‐Gould repair is commonly used, often augmented with an internal brace or suture tape to enhance stability. Advantages: Preserves native anatomy, maintains joint mobility, and has a quicker recovery time. Considerations for Reconstruction Indicated When: Native ligaments are poor quality or deficient. Significant subtalar instability due to injury of the interosseous talocalcaneal ligament or cervical ligament. Recurrence after a prior failed repair. Hypermobility (e.g., generalised ligamentous laxity). High‐demand athletes with multidirectional instability. Techniques: Anatomic reconstruction (using autografts/allografts, such as peroneus longus/brevis or semitendinosus). Non‐anatomic reconstructions (such as the Chrisman‐Snook procedure). Subtalar stabilisation with an arthroereisis implant or subtalar ligament reconstruction. Advantages: Provides stronger and more durable stability, particularly when native ligament quality is inadequate. My Preference If there is isolated lateral ankle instability with good ligament tissue, I prefer a primary repair with augmentation (e.g., Broström‐Gould with suture tape). However, if subtalar instability is significant, I favour reconstruction, particularly if imaging (stress radiographs, MRI, or diagnostic arthroscopy) confirms ligament insufficiency. In cases of subtalar instability, I also evaluate the interosseous talocalcaneal ligament and consider additional procedures such as subtalar ligament reconstruction or subtalar fusion if there is severe instability or degenerative changes. **Subtalar instability** **Q16. How do you assess subtalar instability? Physical examination? What kind of imaging do you perform?** **Answer by experts:** Clinical examination 24 (80.0%) Stress radiographs 13 (40.3%) MRI 8 (26.7%) Arthroscopy 3 (10.0%) Evaluation under anaesthesia 2 (6.7%) Other Stress MRI (1), standing CT (1) Answer by ChatGPT: Assessment of Subtalar Instability 1. Clinical History A thorough history is crucial in assessing subtalar instability. Key points to evaluate include: Recurrent ankle sprains or a feeling of the ankle "giving way." Pain or instability on uneven surfaces. Swelling and tenderness, especially over the sinus tarsi region. Previous trauma, particularly involving the lateral ankle ligaments. 2. Physical Examination Several clinical tests help in assessing subtalar instability: a. Palpation Tenderness over the sinus tarsi (suggests ligament injury). Pain over the calcaneofibular ligament (CFL), which stabilises both the subtalar and talocrural joints. b. Range of Motion (ROM) Assess inversion and eversion of the subtalar joint. Excessive motion compared to the contralateral side suggests instability. c. Stress Tests Medial and Lateral Subtalar Glide Test Stabilise the talus and move the calcaneus medially and laterally. Increased translation compared to the contralateral side suggests instability. Posterior Subtalar Drawer Test Stabilise the tibia and talus while applying a posterior force on the calcaneus. Increased posterior motion may indicate subtalar instability. Tilt Test (Varus and Valgus Stress) Apply a varus/valgus stress to the hindfoot to check for excessive movement. Talar Tilt Test (indirect assessment) Primarily assesses the talocrural joint but may also indicate subtalar instability. 3. Imaging Studies a. X‐rays Standard weight‐bearing X‐rays (AP, lateral, and oblique views) to assess alignment. Stress radiographs (inversion and eversion) may show excessive motion or talar tilt. Canale view (for coalition or fractures). b. CT Scan Best for bony abnormalities, such as subtalar arthritis, calcaneal fractures, or coalition. c. MRI Best for soft tissue evaluation, including the interosseous talocalcaneal ligament (ITCL) and cervical ligament, which are key stabilisers of the subtalar joint. Useful for detecting sinus tarsi syndrome, ligament tears, and tendinopathy. d. Ultrasound Can be used dynamically to assess ligament integrity (e.g., CFL and interosseous ligaments). Summary Physical exam includes palpation, ROM testing, and stress tests (subtalar glide, tilt, and posterior drawer). X‐rays (weight‐bearing and stress views) are first‐line. CT scan is for bony evaluation. MRI is best for soft tissue injuries. Ultrasound can be used dynamically for ligament assessment. Reconstruction of ATFL and CFL? **Q17. If you consider a reconstruction of the ATFL, do you perform a reconstruction of the CFL? Almost always, almost never, it depends on…?** **Answer by experts:** (almost) Always 18 (60.0%) (almost) Never 4 (13.3%) Depends on subtalar instability 6 (20.0%) Depends on CFL quality 3 (10.0%) **Answer by ChatGPT:** The decision to reconstruct the calcaneofibular ligament (CFL) when reconstructing the anterior talofibular ligament (ATFL) depends on several factors, including the patient's degree of ankle instability, clinical findings, imaging results, and intraoperative assessment. General Approach: Almost always: If the patient has chronic ankle instability, both ATFL and CFL are often involved. CFL plays a key role in stabilising the subtalar joint and providing rotational control. If a patient demonstrates significant hindfoot instability, it is common to reconstruct both ligaments. It depends on: Clinical Exam & Imaging: If stress X‐rays (talar tilt >10°) or MRI indicate isolated ATFL insufficiency without CFL involvement, only ATFL reconstruction may be performed. However, if the CFL is lax or functionally deficient, reconstruction is often indicated. Intraoperative Testing: If, after ATFL reconstruction, the ankle still shows instability or excessive subtalar motion (heel inversion instability), the surgeon may opt to reconstruct the CFL. Patient Factors: Athletes or individuals with high‐demand activities may benefit from a combined ATFL‐CFL reconstruction to ensure long‐ term stability. Almost never: In cases of purely isolated ATFL injury with no significant subtalar instability or chronic ankle instability, a standalone ATFL reconstruction may be sufficient. However, isolated injuries without some CFL involvement are less common in chronic instability cases. Summary: If there is combined ATFL and CFL insufficiency → Reconstruct both. If only ATFL is deficient and CFL is functionally intact → ATFL reconstruction alone may be sufficient. Intraoperative testing is often the key determinant.

## DISCUSSION

The most important finding of the present study was that at present ChatGPT‐4 cannot generate answers to clinical queries on the surgical treatment of CLAI equivalent to those of foot and ankle expert. ChatGPT‐4' responses were agreement with the consensus statements for about 65% of the questions. However, ChatGPT‐4 was able to add supplementary information on about 70% of the questions, suggesting its possible utility in the clinical settings of CLAI. The clinical relevance of this study is that improvement is required before ChatGPT can be used reliably by inexperienced or general orthopaedic surgeons when treating patients with CLAI.

Currently, patients frequently use the Internet to obtain medical information [4]. However, the reliability of Internet data is not always guaranteed, and harmful or inaccurate information remains a serious social problem. Recently, Chatbots have been widely used, and the users of ChatGPT had exceeded 100 million within a month of its release [27]. AI‐based Chatbots have been expected as promising tools for providing medical information to patients as well as clinicians [1, 13, 33]. If LLMs can generate reliable information to clinicians such as experts in the field, which would be especially beneficial for inexperienced orthopaedic surgeons.

In the field of orthopaedic surgery, there is an increasing number of studies that have shown the utility of chatbots in delivering and enriching medical information for patients [2, 11, 13, 23]. Some authors have evaluated the feasibility of using ChatGPT to offer reliable information as a reference of clinical practice guidelines [2, 9, 13, 20, 21, 30]. Duey et al. found that 92% of the answers by ChatGPT‐4 for thromboembolic prophylaxis in spine surgery were correct with supplementary information [9]. Yang et al. reported that ChatGPT provided responses that were concordant with the American Academy of Orthopaedic Surgeons CPGs for 60% (12/20) of questions about knee or hip osteoarthritis while encouraging the use of non‐recommended treatments in responses to 30% (6/20) of those queries [30]. The study findings in these studies suggest the utility of ChatGPT for inexperienced or general orthopaedic surgeons in the clinical practice. However, few studies have evaluated the utility of ChatGPT for clinicians as a reference of consensus statements in the field of foot and ankle pathologies.

In the current study, 64.7% (11/17) of the answers by ChatGPT to questions on the surgical treatment of CLAI were agreement with those of the foot and ankle experts, which was similar to the findings of Yang et al. (60%) [30] and by Meija et al. (59%) [21]. Regarding the overconclusiveness, ChatGPT‐4 was overconclusive in 23.5% (4/17) of the questions included in the present study, which was higher than that reported by Duey et al. (8%, 1/12 questions) [9]. However, this value was lower than that reported by Meija et al. (45%, 13/29) [21]. Based on the study findings of these previous studies and the current study, the accuracy of the responses by ChatGPT as a reference for expert opinions is approximately 60%, with the overconclusiveness differing among orthopaedic pathologies. However, ChatGPT was able to provide supplementary information on 70.6% of the questions and the incompleteness of its answers was 11.2%. Accordingly, given that many surgical issues still remain a matter of debate [5, 7, 26], LLMs would have a supplementary role when non‐expert surgeons treat patients with CLAI. An inherent shortcoming of ChatGPT is the generation of responses based on data learned from diverse sources without specific emphasis on more recent high‐quality evidence, which is associated with the risk of providing incorrect and outdated data to patients and clinicians [19, 29]. Although chatbots have dramatically contributed to medical advancement and convenience, continuous updating of research data and establishing a way of generating evidence‐based responses by chatbots are needed in order to apply chatbots to orthopaedic surgery as a really reliable and powerful tool. Strategies such as retrieval augmentation and fine‐tuning with human feedback would result in generating more accurate and reliable responses to clinical questions [17, 34].

There were several limitations to the present study. First, consensus statements and answers by experts in this study were developed in 2017 [22]. Accordingly, these statements potentially may not be updated and may change once lately published studies are incorporated into future versions. However, this limitation has also been found in previous studies with similar study designs [9, 18, 20, 30]. Second, the specific iteration of the ChatGPT model may have influenced the study outcomes. Emerging models optimised for complex reasoning can produce responses with greater clinical accuracy compared to ChatGPT‐4. Furthermore, the input methodology does not incorporate advanced prompting strategies. It is conceivable that leveraging structured prompt engineering, such as instructing the model to simulate the clinical reasoning of an orthopaedic surgeon advising a peer, could have yielded qualitatively different responses. However, Slawaska‐Eng et al. reported no significant difference in performance between ChatGPT‐3.5 and ‐4 when answering questions on femoroacetabular impingement [28]. The significant finding of this study was that ChatGPT‐4 was not able to generate a comparative answer to the consensus statements developed in 2017, although ChatGPT‐4 had learned more recent data after 2017. Third, although some questions such as Q7 and Q8 that were answered wrong by ChatGPT, these questions are still highly controversial among experts. Fourth, this study used only 17 questions about the surgical treatment of CLAI, limiting the ability to investigate the answers by ChatGPT to questions regarding the diagnosis or return to sports after CLAI surgery. Fourth, consistency of the ChatGPT's responses in different sessions or users was not considered in this study. Finally, the current study did not evaluate the accuracy of answers by previous versions of ChatGPT or LLMs other than ChatGPT. Despite failing to completely match the consensus statements, the study findings indicate the potential utility of ChatGPT in patient education and for non‐expert clinicians in clinical settings. However, cautious application and further evaluation are recommended considering the limitations of the present study and the potential enhancement of the possibility of LLMs that were not verified in this study.

## CONCLUSION

The present study showed that ChatGPT‐4 was not able to provide answers to queries on the surgical treatment of CLAI such as experts of the ESSKA‐AFAS AIG. ChatGPT‐4's answers were agreement with the consensus statements for 64.7% of the questions. However, ChatGPT also showed its promising potential for its application to inexperienced or general orthopaedic surgeons when treating patients with CLAI.

## AUTHOR CONTRIBUTIONS

Hélder Pereira and Takuji Yokoe conceptualised this study. Takuji Yokoe, Giulia Roversi and Hélder Pereira evaluated the responses by ChatGPT. Takuji Yokoe and Hélder Pereira mainly drafted the manuscript, and Pedro Diniz, Nuno Sevivas, and Naosuke Kamei supervised it. All authors had complete access to all data used in this study and take responsibility for its accuracy. All authors have read and approved the final manuscript.

## CONFLICT OF INTEREST STATEMENT

The authors declare no conflicts of interest.

## ETHICS STATEMENT

The institutional review board approval was waved for this study because no human or animal subjects were included.

## Data Availability

All relevant data analysed in this study is included in this article. Further inquiries can be directed to the corresponding author.

## References

[1] Abbasgholizadeh Rahimi S , Légaré F , Sharma G , Archambault P , Zomahoun HTV , Chandavong S , et al. Application of artificial intelligence in community‐based primary health care: systematic scoping review and critical appraisal. J Med Internet Res. 2021;23(9):e29839. 34477556 10.2196/29839 PMC8449300

[2] Ayık G , Ercan N , Demirtaş Y , Yıldırım T , Çakmak G . Evaluation of ChatGPT‐4o's answers to questions about hip arthroscopy from the patient perspective. Joint Dis Related Surg. 2025;36(1):193–199. 10.52312/jdrs.2025.1961 PMC11734852 39719917

[3] Brown AJ , Shimozono Y , Hurley ET , Kennedy JG . Arthroscopic versus open repair of lateral ankle ligament for chronic lateral ankle instability: a meta‐analysis. Knee Surg Sports Traumatol Arthrosc. 2020;28(5):1611–1618. 30109370 10.1007/s00167-018-5100-6

[4] Calixte R , Rivera A , Oridota O , Beauchamp W , Camacho‐Rivera M . Social and demographic patterns of health‐related internet use among adults in the United States: a secondary data analysis of the Health Information National Trends Survey. Int J Environ Res Public Health. 2020;17(18):6856. 32961766 10.3390/ijerph17186856 PMC7559701

[5] Cho BK , Kim SH , Choi SM , Hwang ET . Usefulness of suture‐tape augmentation based on intraoperative ankle stress radiographs during anatomical ligament repair for chronic lateral ankle instability. Foot Ankle Int. 2025;46(1):54–63. 39560141 10.1177/10711007241291049

[6] Dahmen J , Kayaalp ME , Ollivier M , Pareek A , Hirschmann MT , Karlsson J , et al. Artificial intelligence bot ChatGPT in medical research: the potential game changer as a double‐edged sword. Knee Surg Sports Traumatol Arthrosc. 2023;31(4):1187–1189. 36809511 10.1007/s00167-023-07355-6

[7] Dallman J , Wolf MR , Campbell T , Herda T , White J , Tarakemeh A , et al. Current definitions of failure in lateral ankle instability surgery: a systematic review. Am J Sports Med. 2023;51(10):2748–2757. 36917833 10.1177/03635465231153165

[8] Doherty C , Bleakley C , Hertel J , Caulfield B , Ryan J , Delahunt E . Recovery from a first‐time lateral ankle sprain and the predictors of chronic ankle instability. Am J Sports Med. 2016;44(4):995–1003. 26912285 10.1177/0363546516628870

[9] Duey AH , Nietsch KS , Zaidat B , Ren R , Ndjonko LCM , Shrestha N , et al. Thromboembolic prophylaxis in spine surgery: an analysis of ChatGPT recommendations. The Spine Journal. 2023;23(11):1684–1691. 37499880 10.1016/j.spinee.2023.07.015

[10] Feng SM , Sun QQ , Xue C , Maffulli N , Oliva F , Luo X . Arthroscopic lateral ligament reconstruction for isolated chronic lateral ankle instability is associated with longer recovery compared to arthroscopic Broström repair and inferior extensor retinaculum augmentation. Injury. 2025;56(2):112082. 39700785 10.1016/j.injury.2024.112082

[11] Hu X , Niemann M , Kienzle A , Braun K , Back DA , Gwinner C , et al. Evaluating ChatGPT responses to frequently asked patient questions regarding periprosthetic joint infection after total hip and knee arthroplasty. Digit Health. 2024;10:20552076241272620. 10.1177/20552076241272620 39130521 PMC11311159

[12] Hu Y , Li Q , Li X , Xie Y , Liu C , Fu C , et al. Evaluation of open versus arthroscopic anterior talofibular ligament reconstruction for chronic lateral ankle instability with talar and subtalar cartilage MRI T2 mapping: a 3‐year prospective study. Am J Sports Med. 2024;52(3):730–738. 38305002 10.1177/03635465231222931

[13] Kaarre J , Feldt R , Keeling LE , Dadoo S , Zsidai B , Hughes JD , et al. Exploring the potential of ChatGPT as a supplementary tool for providing orthopaedic information. Knee Surg Sports Traumatol Arthrosc. 2023;31(11):5190–5198. 37553552 10.1007/s00167-023-07529-2 PMC10598178

[14] Kaarre J , Feldt R , Zsidai B , Senorski EH , Rydberg EM , Wolf O , et al. ChatGPT can yield valuable responses in the context of orthopaedic trauma surgery. J Exp Orthop. 2024;11(3):e12047. 38887661 10.1002/jeo2.12047 PMC11180970

[15] Kuşcu O , Pamuk AE , Sütay Süslü N , Hosal S . Is ChatGPT accurate and reliable in answering questions regarding head and neck cancer? Front Oncol. 2023;13:1256459. 38107064 10.3389/fonc.2023.1256459 PMC10722294

[16] Landis JR , Koch GG . The measurement of observer agreement for categorical data. Biometrics. 1977;33(1):159–174. 843571

[17] Li J , Dada A , Puladi B , Kleesiek J , Egger J . ChatGPT in healthcare: a taxonomy and systematic review. Comput Methods Programs Biomed. 2024;245:108013. 38262126 10.1016/j.cmpb.2024.108013

[18] Liang Z , Wang M , Abdelatif NMN , Arunakul M , Borbon CAV , Chong KW , et al. Are large language model‐based chatbots effective in providing reliable medical advice for achilles tendinopathy? An international multispecialist evaluation. Orthop J Sports Med. 2025;13(4):23259671251332596. 40322749 10.1177/23259671251332596 PMC12046157

[19] Longwell JB , Hirsch I , Binder F , Gonzalez Conchas GA , Mau D , Jang R , et al. Performance of large language models on medical oncology examination questions. JAMA Netwk Open. 2024;7(6):e2417641. 10.1001/jamanetworkopen.2024.17641 PMC11185976 38888919

[20] Magruder ML , Rodriguez AN , Wong JCJ , Erez O , Piuzzi NS , Scuderi GR , et al. Assessing ability for ChatGPT to answer total knee arthroplasty‐related questions. J Arthroplasty. 2024;39(8):2022–2027. 38364879 10.1016/j.arth.2024.02.023

[21] Mejia MR , Arroyave JS , Saturno M , Ndjonko LCM , Zaidat B , Rajjoub R , et al. Use of ChatGPT for determining clinical and surgical treatment of lumbar disc herniation with radiculopathy: a North American Spine Society guideline comparison. Neurospine. 2024;21(1):149–158. 38291746 10.14245/ns.2347052.526 PMC10992643

[22] Michels F , Pereira H , Calder J , Matricali G , Glazebrook M , Guillo S , et al. Searching for consensus in the approach to patients with chronic lateral ankle instability: ask the expert. Knee Surg Sports Traumatol Arthrosc. 2018;26(7):2095–2102. 28439639 10.1007/s00167-017-4556-0

[23] Mika AP , Martin JR , Engstrom SM , Polkowski GG , Wilson JM . Assessing ChatGPT responses to common patient questions regarding total hip arthroplasty. J Bone Jt Surg. 2023;105(19):1519–1526. 10.2106/JBJS.23.00209 37459402

[24] Oeding JF , Lu AZ , Mazzucco M , Fu MC , Taylor SA , Dines DM , et al. ChatGPT‐4 performs clinical information retrieval tasks using consistently more trustworthy resources than does Google search for queries concerning the latarjet procedure. Arthroscopy. 2025;41(3):588–597. 38936557 10.1016/j.arthro.2024.05.025

[25] Ollivier M , Pareek A , Dahmen J , Kayaalp ME , Winkler PW , Hirschmann MT , et al. A deeper dive into ChatGPT: history, use and future perspectives for orthopaedic research. Knee Surg Sports Traumatol Arthrosc. 2023;31(4):1190–1192. 36894785 10.1007/s00167-023-07372-5

[26] Pereira H , Vuurberg G , Spennacchio P , Batista J , D'Hooghe P , Hunt K , et al. Surgical treatment paradigms of ankle lateral instability, osteochondral defects and impingement. Adv Exp Med Biol. 2018;1059:85–108. 29736570 10.1007/978-3-319-76735-2_4

[27] Shahsavar Y , Choudhury A . User intentions to use ChatGPT for self‐diagnosis and health‐related purposes: cross‐sectional survey study. JMIR Hum Factors. 2023;10:e47564. 37195756 10.2196/47564 PMC10233444

[28] Slawaska‐Eng D , Bourgeault‐Gagnon Y , Cohen D , Pauyo T , Belzile EL , Ayeni OR . ChatGPT‐3.5 and ‐4 provide mostly accurate information when answering patients’ questions relating to femoroacetabular impingement syndrome and arthroscopic hip surgery. J ISAKOS. 2025;10:100376. 39674512 10.1016/j.jisako.2024.100376

[29] Truhn D , Reis‐Filho JS , Kather JN . Large language models should be used as scientific reasoning engines, not knowledge databases. Nature Med. 2023;29(12):2983–2984. 37853138 10.1038/s41591-023-02594-z

[30] Yang J , Ardavanis KS , Slack KE , Fernando ND , Della Valle CJ , Hernandez NM . Chat Generative Pretrained Transformer (ChatGPT) and Bard: artificial intelligence does not yet provide clinically supported answers for hip and knee osteoarthritis. J Arthroplasty. 2024;39(5):1184–1190. 38237878 10.1016/j.arth.2024.01.029

[31] Yapar D , Demir Avcı Y , Tokur Sonuvar E , Faruk Eğerci Ö , Yapar A . ChatGPT's potential to support home care for patients in the early period after orthopedic interventions and enhance public health. Joint Dis Relat Surg. 2024;35(1):169–176. 10.52312/jdrs.2023.1402 PMC10746912 38108178

[32] Yokoe T , Tajima T , Chosa E , Yamaguchi N , Morita Y . Screening of undiagnosed increased lateral ankle laxity using stress ultrasonography. Orthop J Sports Med. 2024;12(4):23259671241235162. 38571485 10.1177/23259671241235162 PMC10986172

[33] Yu KH , Healey E , Leong TY , Kohane IS , Manrai AK . Medical artificial intelligence and human values. N Engl J Med. 2024;390(20):1895–1904. 38810186 10.1056/NEJMra2214183 PMC12425466

[34] Zakka C , Chaurasia A , Shad R , Dalal AR , Kim JL , Moor M , et al. Almanac: retrieval‐augmented language models for clinical medicine. NEJM AI. 2024;1(2):10.1056. 10.1056/aioa2300068 PMC10857783 38343631

[35] Zsidai B , Kaarre J , Hilkert AS , Narup E , Senorski EH , Grassi A , et al. ESSKA Artificial Intelligence Working Group Accelerated evidence synthesis in orthopaedics‐the roles of natural language processing, expert annotation and large language models. J Exp Orthop. 2023;10(1):99. 37768352 10.1186/s40634-023-00662-4 PMC10539226

